# Wild and backyard food use during COVID-19 in upstate New York, United States

**DOI:** 10.3389/fnut.2023.1222610

**Published:** 2023-09-05

**Authors:** Jeanne L. Coffin-Schmitt, Nia Clements, Grace Marshall, Lu Liu, Aly Trombitas, Zi Wang, Shuai Yuan, Amelia Greiner Safi, Karla L. Hanson, Kathryn J. Fiorella

**Affiliations:** ^1^Department of Natural Resources and Environment, Cornell University, Ithaca, NY, United States; ^2^Department of Public and Ecosystem Health, Cornell University, Ithaca, NY, United States

**Keywords:** hunting, fishing, foraging, backyard poultry, gardening, food security, pandemic, food choice

## Abstract

**Introduction:**

COVID-19 acutely shocked both socio-economic and food systems in 2020. We investigated the impact of COVID-19 on production and consumption of gardened produce, backyard poultry, wild game and fish, and foraged mushrooms, berries, and other plants in New York State, aiming to understand crisis influenced food choice and motivations, including food security.

**Methods:**

We conducted an online, cross-sectional survey in October–December 2020 with a convenience sample of participants (*n* = 505) with an interest in gardening, poultry rearing, foraging, hunting, and/or fishing from six counties in upstate New York. We recruited through the New York Department of Environmental Conservation, Cornell Cooperative Extension, and other relevant email and social media pages.

**Results:**

Across the wild and backyard food production strategies, 4.0–14.3% of respondents reported engaging for the first time and 39.6–45.7% reported increased production (a little or a lot more), and 31.6–42.7% of respondents’ production was the same as the previous year. Consumption of foods produced was widespread, including fruit and vegetables (97.6% of producers also consumed), backyard eggs (92.7%), and foraged foods (93.8%). For meats, a majority consumed backyard poultry meat (51.2%), wild-caught fish (69.7%), and wild game they hunted (80.1%). The frequency of consumption of fruit and vegetables (average of 13.5 times/month) and eggs (16.4 times/month) was very high, while average consumption of poultry meat, foraged foods, fish, and wild game ranged from 3.1 to 5.8 times/month. The number of respondents who reported “have more control over food availability” as motivation to produce all wild and backyard foods increased from 2019 to 2020 (*p* < 0.05 - *p* < 0.001). There was also a significant relationship between experiences of COVID-19 related hardship (i.e., food insecurity, income loss) with gardening and poultry-rearing (*p* ≤ 0.05), but not with other production methods or with consumption of wild and backyard foods.

**Discussion:**

Our findings help to locate wild and backyard foods within COVID-19 impacted food environments, and describe food security as a particularly relevant motivation, among others, reported by respondents in 2020. Given this, New York State service providers can use these findings to tailor current future support for households exerting control over their own food environments with wild and backyard foods, allowing the state to be better prepared for future crises.

## Introduction

The COVID-19 pandemic constituted a global shock, with far-reaching, interconnected impacts on global socio-economic and food systems beginning in 2020 (1). Lost jobs, cut hours, and furloughs impacted incomes and well-being (2). At the same time, food supply chains faltered amid soaring demand for nonperishable, low-cost foods; forced closures of restaurants; and challenges in providing COVID-safe working conditions (3, 4). These parallel economic and food system impacts in early 2020 acutely shifted global food environments. For households and individuals, food choices had to be made in the face of uncertainty about food availability due to unstable supply chains, as well as variable lock down orders, income changes, and food safety concerns. Consequent shifts in eating behavior have been observed around the world, with diet quality largely found to have worsened during the early pandemic (5). Global food insecurity worsened precipitously (6, 7).

Here we examined how early responses to the COVID-19 pandemic shifted food environments and food consumption patterns. We used the case of New York State in 2020 to analyze production and consumption of wild and backyard food in a food environment stressed by a large-scale crisis.

### Wild and backyard foods use expanded during COVID-19

To contextualize our analysis, we review the literature regarding shifts in wild and backyard food production early in the pandemic. We include geographies around the world, but emphasize United States, Canadian, and European settings most like our study site in upstate New York, United States.

Within the altered food environments and public health lockdown restrictions, wild and backyard food production – including gardening, raising backyard poultry, foraging, fishing, and hunting – was reported both as a pastime and a food access strategy (8, 9). Reports of widespread engagement in backyard gardening and poultry production, as well as upticks in use of private and wild lands for foraging, hunting, and fishing were soon widespread across the United States, Canada, Europe and other Global North settings (10–12). Alongside these shifts came calls for a resurgence of victory gardens, stock-outs of seeds and tomato cages, and unprecedented demand for hunting and fishing licenses (8, 9, 13).

Researchers found shifting roles and growth in gardening, a range of food safety concerns stemming from backyard poultry expansion, high participation in hunting and fishing, and isolated reports of increased foraging. In contrast to a growing literature on participation in wild and backyard food *production,* very limited information ties these activities to shifts in food *consumption patterns* or other health outcomes, though we incorporate these findings wherever available.

The early stages of the pandemic saw widespread proliferation of gardening. A global analysis of search terms showed that online interest in gardening (from Google Trends data) was strongly synchronized with the initial waves of infection (14). In Louisiana (United States), 82% gardeners responding to a consumer survey increased gardening effort (15), and in Kentucky (United States), increases in gardening were linked directly to changing food environments (16). In other Global North settings, similar trends were observed. In Canada, 17.4% of gardeners surveyed began gardening in 2020 (17). In parts of Europe, there was an approximately 10% increase in all types of home gardening (18). A study in Taiwan found that pandemic-related stress indirectly promoted intentions to garden (19).

Gardeners and community garden support organizations also reported facing pandemic-related barriers (20–22). In the early pandemic, survey respondents across the Global North reported more challenges from COVID-19 and put a higher value on gardening to produce food and save money (20). However, pandemic-related reasons for gardening or not gardening were highly variable depending on age, gardening experience, and time at home in a 2020–2021 survey in the United States (23). This indicates that access to gardening as a pandemic coping strategy was far from universal, despite its desirability and usefulness, in agreement with studies in diverse locations from Europe to Benin to China (24–26).

Many studies showed pandemic gardening was related to physical, mental, and social well-being. Pandemic gardening supported access to healthy food, spaces for creativity, and created safe and positive social connections in worldwide locations (20, 22, 27). In urban upstate New York, it facilitated connection with other gardeners and nature, supported mental and physical well-being, and contributed to community resilience efforts (21). Increased gardening was spurred by ethos around seeking well-being and self-sufficiency for communities at risk of poor food access in Italy and in Arizona, United States (28, 29). In Benin, a study found that access to gardens protected food security in rural and urban areas from pandemic associated impacts (24). Several studies found a widespread perception that gardening mitigated pandemic-related stress from lockdowns in parts of Europe, North and South America, Australia, China, and Taiwan (19–21, 26, 30–33).

A few studies used established measures to assess the health of gardeners. Gardening showed a generalized protective effect on the Depression, Anxiety, and Stress Scale (21-item) in India (34). In the United States, gardening showed a stronger protective effect on Generalized Anxiety Disorder (7-item) scores for experienced gardeners while age, geographical location, and gender also moderated the protective effect (35). However, in Scotland, while gardening was associated with self-reported improvements in health, it was not associated with better health outcomes such as body mass index, anxiety, depression, diabetes, or cardiovascular disease (36).

While we found little evidence on the extent of backyard poultry production shifts and who participated, expansions in backyard poultry production have quickly given way to concerns about disease transmission for new producers. In Vermont, production of backyard chickens was most common in rural areas and among wealthier and more educated households (37). Larsen et al., also showed low uptake of biosecurity practices among backyard poultry rearers, and that nearly 20% of backyard flocks have *Salmonella enterica*. Across the United States, 2020 saw more than 1,700 Salmonella outbreaks linked to privately owned poultry, an increase over previous years driven by COVID-19 pandemic engagement in poultry rearing (38).

Hunting participation generally increased during the pandemic. A survey of wildlife biologists across the United States revealed that during turkey hunting season 2020 many states saw an increase in hunting license sales, hunting effort, and harvest compared to the mean from the previous 3 years; this change was not due to an increase in turkey abundance (13). In another example, while non-resident turkey permits were closed in Nebraska to discourage travel, the number of resident hunters increased by 23% and resident permits rose by 26% (39). In Calakmul region, Campeche, Mexico, twice as much hunting effort and take of white tail deer was observed during mid 2020 (40).

Fishing within traditionally ‘recreational’ fisheries (41) largely increased during the early phases of the pandemic. Although in several surveys anglers noted access barriers at some points during COVID-19 (42–44), the COVID-19 lockdown policies of more than 90% of United States and Canadian provinces ultimately permitted recreational fishing (45). In many settings, fishing flourished. Over a quarter of respondents in an European survey reported increasing fishing trips (46), and anglers credited fishing as an important support to their mental health. In a survey across ten United States, the number of trips per angler significantly increased (43). In a survey of recreational fishers in Ontario (Canada), 21% of respondents said they had resumed fishing or newly began fishing (47) and radio telemetry showed an 8-fold increase in exploitation rate (48). In Wisconsin (United States), in-state license sales increased a striking 71% and lakes with public shorelines saw increased visitors (49).

While limited evidence about shifts in foraging specifically has emerged, those studies that address it did see changes. One study found that during the pandemic foraging was integrated into urban food provisioning strategies (50), while another found that all outdoor activities (including gardening and foraging) increased (51).

### Impacts of COVID-19 on New York state

While the pandemic also impacted policies and supply chains in ways that affected access to wild and backyard foods, locales largely permitted these activities (45). The few studies of shifting participation in wild and backyard food production have shown growth in participation in the early phases of the pandemic. A representative survey in Vermont found over a third of households participated in wild and backyard food production, with half of participants engaging for the first time or more intensely as a result of the pandemic (52). Yet, while food insecure households more intensely produced wild and backyard foods, only food secure households saw higher fruit and vegetable consumption from these sources (52).

The specific context of New York state’s COVID-19 timeline and control measures affected its wider food environment, as well as access to wild and backyard food production during the study period. A state of emergency was declared March 7, 2020. By the end of March, New York State was ‘On Pause’ with non-essential workers at home and schools closed. Residents were told to stay home. All events were canceled. Over 2 months later, on May 15, 2020, New York implemented a phased re-opening of non-essential businesses. Each region within the state was assigned one of four phases weekly, depending on the COVID-19 health metrics at that time. This study was conducted in counties that are part of three New York regions, but those regions had similar trajectories through the phase system; individuals in the studied counties largely experienced similar restrictions at any given time. For example, all 6 counties were assigned to phase one on May 15, 2020, and to phase two by May 28, 2020.

The relevant restrictions for wild and backyard food production were as follows: in phase one individual fishing and hunting was allowed by the state, contingent on any additional guidance of local governing bodies (i.e., cities, municipalities). However, commercial fishing services and for-hire fishing vessels were required to follow state-level public health guidelines (53). Indoor retail stores selling gardening, poultry-rearing, fishing, and hunting supplies reopened in phase two.

Wild and backyard food production in New York is supported by several different local service providers which host a variety of educational, licensing, and supportive services. Hunting and fishing are administered by the New York Department of Environmental Conservation in collaboration with the New York Department of Health; both have an in-depth but sometimes difficult to navigate online presence. Cornell Cooperative Extension acts at county and state level, supporting gardening and poultry raising at large and small scales, as well as agroforestry. Its county offices host detailed and locally variable educational resources online, and sometimes have social media presences as well. Education and support for foraging, however, is largely decentralized and privately run for profit.

When “NY On Pause” was initiated, all in-person educational events were canceled. This included hunter education classes, gardening classes, fishing promotion events, foraging education courses, expert consultations for troubleshooting, and more. However, fishing and hunting licenses were consistently available throughout for online purchase. Beginning April 15, 2020, the New York hunter education certificate became fully available online, removing the requirement for in-person classes. In general, service providers had to lean on whatever previously developed remote and online resources they had at first, and then adjust to each change in policy as they were rolled out.

This work is guided by two primary research questions: (1) how did production and consumption of wild and backyard foods shift during the early months of the COVID-19 pandemic, (2) do associations exist between food insecurity and the production and consumption of wild and backyard foods. These findings have implications for food access and well-being during a time of acute systemic stress on food environments and may assist local and regional wild and backyard food service providers and support organizations to help individuals and households cope better in the future.

## Materials and methods

We conducted an online cross-sectional survey with a convenience sample of upstate New York residents (*n* = 505). We chose six counties (Broome, Cortland, Onondaga, Oswego, Cayuga, and Seneca counties) that provided local opportunities for all five food production activities and encompassed both rural and urban areas within central and upstate New York. The survey was open between October 26 – December 10, 2020. The study was exempted from IRB review by the Cornell IRB (Protocol ID#: 2008009765).

### Survey distribution and eligibility requirements

Cornell Cooperative Extension offices in each of the six counties and the New York Department of Environmental Conservation supported recruitment of adults with an interest in gardening, poultry rearing, foraging, hunting, and/or fishing. Cornell Cooperative Extension offices shared information about the survey through their websites, relevant email lists (e.g., 4-H, Volunteer Network), and social media presence (e.g., Facebook and Twitter for Cornell Cooperative Extension-Broome County). The New York Department of Environmental Conservation distributed the survey through their Hunting, Fishing, Sustainability, and Becoming an Outdoors-Woman email lists. In addition, the survey was distributed through social media community groups and message boards in relevant topics (i.e., the page for a town in the sample area, an Upstate New York hunting and fishing group, Central New York Gardeners, etc.), mutual aid groups, and a local newspaper.^1^ This resulted in a convenience sample of adult residents who were likely to participate in food self-provisioning activities. Respondents were offered a chance to opt into a raffle to win one of 20 gift cards for $50 to a local grocery store for survey participation. Identifying information was kept separately from analyzed data and stored per Cornell IRB’s requirements. This online sample aimed to capture adults who participate in the production and consumption of wild and backyard foods. The survey was conducted online to reduce COVID-19 risks for respondents. The respondent population is non-random and therefore biased, including, for example, towards those who learned of the survey, had online access, and were available and interested to take the survey. We compared survey respondent demographic proportions to the 2020 census numbers to assess the bias introduced.

### Survey domains

All respondents answered questions in domains covering demographics and COVID-19 impacts on employment (Table 1) and general food procurement (Table 2) from the National Food Access and COVID-19 Research Team (6, 54). This module asked about 7 types of food assistance and 11 sources of food being used at the time of survey, with a parallel reference period of ‘the same time period in 2019’. We also captured COVID-19-related impacts on employment using the same approach. Demographic characteristics included education level, income, gender, race, and ethnicity. Food security was assessed with the U.S. Department of Agriculture’s Household Food Security Survey Module: Six-Item Short Form over a 30-day reference period (55) again with the parallel reference period in 2019 (Table 2).

**Table 1:** Respondent demographics overall and by food self-procurement activity during the COVID-19 outbreak.

	All households *n* (%)	Gardening *n* (%)	Poultry *n* (%)	Foraging *n* (%)	Fishing *n* (%)	Hunting *n* (%)
Gender	*n* = 431	*n* = 289	*n* = 40	*n* = 110	*n* = 119	*n* = 136
Male	136 (31.6)	83 (28.7)	10 (25.0)	41 (37.3)	60 (50.4)	76 (55.9)
Female	274 (63.6)	190 (65.7)	28 (70.0)	64 (58.2)	57 (47.9)	54 (39.7)
Prefer not to answer	16 (3.7)	13 (4.5)	1 (2.5)	4 (3.6)	2 (1.7)	6 (4.4)
Self-describe	5 (1.2)	3 (1.0)	1 (2.5)	1 (0.9)	0 (0.0)	0 (0.0)
Income	*n* = 412	*n* = 278	*n* = 40	*n* = 109	*n* = 116	*n* = 130
<$15,000	15 (3.6)	9 (3.2)	1 (2.5)	6 (5.5)	3 (2.6)	2 (1.5)
$15,000 to $24,999	28 (6.8)	18 (6.5)	3 (7.5)	6 (5.5)	4 (3.4)	6 (4.6)
$25,000 to $49,999	78 (18.9)	49 (17.6)	4 (10.0)	17 (15.6)	21 (18.1)	24 (18.5)
$50,000 to $74,999	96 (23.3)	70 (25.2)	10 (25.0)	32 (29.4)	30 (25.9)	34 (26.2)
$75,000 to $149,999	139 (33.7)	93 (33.5)	17 (42.5)	35 (32.1)	42 (36.2)	47 (36.2)
$150,000+	56 (13.6)	39 (14.0)	5 (12.5)	13 (11.9)	16 (13.8)	17 (13.1)
Education	*n* = 431	*n* = 289	*n* = 40	*n* = 110	*n* = 119	*n* = 136
Some high school (no diploma)	1 (0.2)	0 (0.0)	0 (0.0)	0 (0.0)	0 (0.0)	0 (0.0)
High school graduate (including GED)	22 (5.1)	13 (4.5)	2 (5.0)	5 (4.5)	4 (3.4)	9 (6.6)
Some college (no degree)	63 (14.6)	43 (14.9)	8 (20.0)	24 (21.8)	24 (20.2)	21 (15.4)
Associate degree/technical school/apprenticeship	77 (17.9)	48 (16.6)	8 (20.0)	21 (19.1)	28 (23.5)	33 (24.3)
Bachelor’s degree	131 (30.4)	91 (31.5)	11 (27.5)	31 (28.2)	33 (27.7)	44 (32.4)
Postgraduate (Master’s, PhD) or professional degree (JD)	137 (31.8)	94 (32.5)	11 (27.5)	29 (26.4)	30 (25.2)	29 (21.3)
Ethnicity	*n* = 426	*n* = 286	*n* = 40	*n* = 109	*n* = 117	*n* = 134
Not of Hispanic/Latino/Spanish origin	415 (97.4)	278 (97.2)	37 (92.5)	106 (97.2)	112 (95.7)	131 (97.8)
Hispanic—Puerto Rican	6 (1.4)	6 (2.1)	3 (7.5)	3 (2.8)	4 (3.4)	3 (2.2)
Hispanic—Another origin (Self-describe)	5 (1.2)	2 (0.7)	0 (0.0)	0 (0.0)	1 (0.9)	0 (0.0)
Race	*n* = 430	*n* = 289	*n* = 40	*n* = 110	*n* = 119	*n* = 136
American Indian/Alaska Native	7 (1.6)	4 (1.4)	1 (2.5)	3 (2.7)	5 (4.2)	4 (2.9)
Asian/Asian American	6 (1.4)	4 (1.4)	0 (0.0)	1 (0.9)	0 (0.0)	0 (0.0)
Black/African American	5 (1.2)	4 (1.4)	1 (2.5)	1 (0.9)	2 (1.7)	1 (0.7)
Hispanic/Latinx/Spanish origin	9 (2.1)	8 (2.8)	3 (7.5)	3 (2.7)	5 (4.2)	3 (2.2)
Middle Eastern/North African	2 (0.5)	2 (0.7)	0 (0.0)	0 (0.0)	0 (0.0)	0 (0.0)
Native Hawaiian/Pacific Islander	3 (0.7)	2 (0.7)	1 (2.5)	1 (0.9)	1 (0.8)	1 (0.7)
White	393 (91.4)	260 (90.0)	37 (92.5)	102 (92.7)	109 (91.6)	124 (91.2)
Prefer not to answer	16 (3.7)	11 (3.8)	0 (0.0)	2 (1.8)	2 (1.7)	4 (2.9)
Self-describe	10 (2.3)	9 (3.1)	1 (2.5)	4 (3.6)	3 (2.5)	7 (5.1)

**Figure 1:**
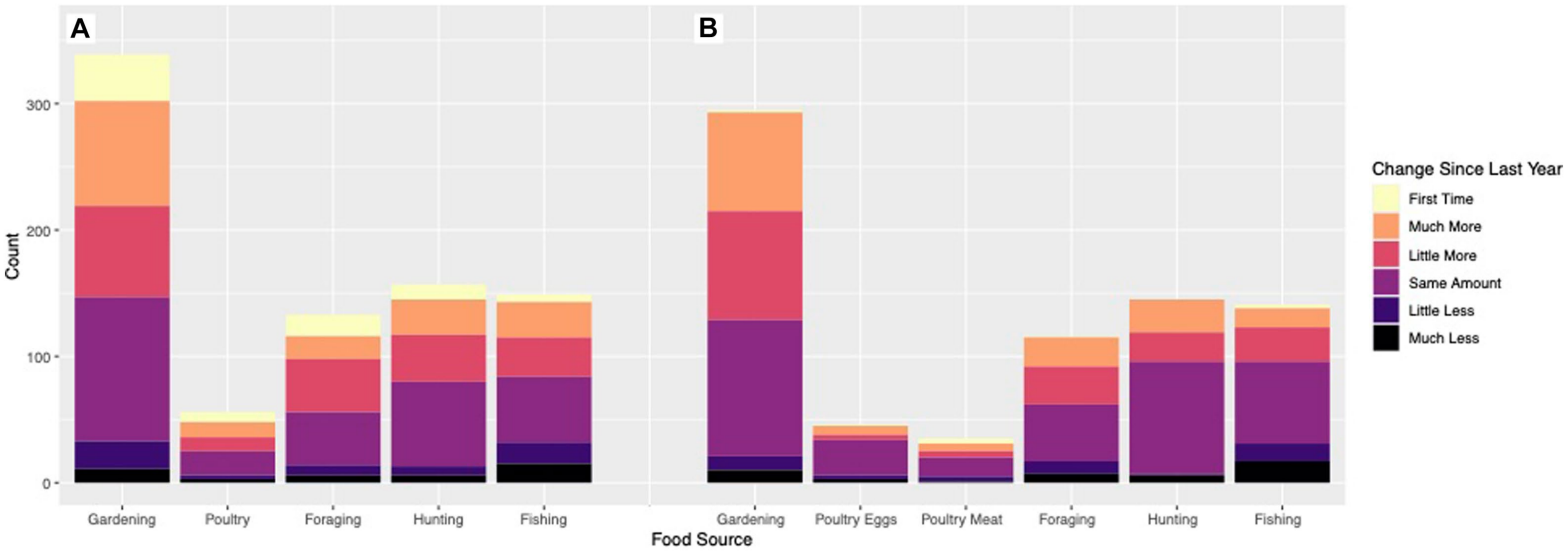
Change in **(A)** production and **(B)** consumption of wild and backyard foods.

To assess COVID-19 impact on general food procurement and production and consumption of wild and backyard foods, we referred in the survey to the “pre-COVID” period as “2019.” This was compared to the period “since the COVID-19 outbreak,” defined as the time between March 2020 and late fall 2020 (October–December), when the survey was conducted. General food procurement investigated *purchased food* (such as grocery shopping options, delivery options, restaurant options, local and alternative options) and *food assistance* (including federal programs like the Supplemental Nutrition Assistance Program or SNAP, formerly food stamps and the Special Supplemental Nutrition Program for Women, Infants, and Children or WIC; local programs such as community food closets and food banks; food or money for food from family, friends, or neighbors; and religious community support). Perceived shifts in production effort of wild and backyard foods (Figure 1) were assessed by asking which of six ordinal choices best described their effort in 2020, compared with 2019 (choices: ‘first time’, ‘much more’, ‘a little more’, ‘the same amount’, ‘a little less’, and ‘a lot less’). Participants were asked where they gardened, reared poultry, foraged, hunted, and fished but these answers are not reported here. Wild and backyard food production effort and consumption were assessed based on the below definitions at the group level. Changes in use of individual types of food within each group were not assessed.

**Table:** 

Definitions of production methods in online survey	
Gardening	Vegetables, fruits, and herbs
Backyard poultry rearing	Eggs and meat
Foraging	Berries or other fruit, greens or other vegetables, roots, mushrooms, medicinal plants
Hunting	White tailed deer, waterfowl, turkey, upland birds such as grouse, small game, and other
Fishing	Cold water fish such as salmon or trout, etc. and warm water fish such as bass, catfish, perch, sunfish, walleye, etc.

To assess food consumption changes (Table 3), we used a question developed for the National Food Access and COVID-19 Research Team survey to measure changes in fruit and vegetable and red and processed meat consumption during the COVID-19 pandemic as compared to the previous year (6). We also adapted a food frequency questionnaire to specifically focus on frequency of consumption of fish, game, fruits, vegetables, poultry, and eggs, and identify the source of these foods (e.g., wild or backyard production; from family, friends or neighbors; purchased from a farm; purchased from a store). Among those who produced wild and backyard foods, we asked how their consumption had changed. Perceived shifts in consumption of wild and backyard foods were assessed by asking which of six ordinal choices best described their consumption in 2020, compared with 2019 (choices: ‘first time’, ‘much more’, ‘a little more’, ‘the same amount’, ‘a little less’, and ‘a lot less’).

Respondents who indicated they practiced gardening, backyard poultry, foraging, hunting, and/or fishing were asked questions about their practices, effort, skill levels, and challenges regarding each activity. Respondents were asked to compare 2019 and 2020. As the popular deer hunting season was beginning at the time of our survey (October–December), hunting was addressed by asking about respondents’ 2020 hunting plans. For other activities, respondents were asked retrospectively about the recent production season and comparisons to 2019. All respondents answered a set of questions regarding motivations for why they chose to get food they produced or harvested themselves by gardening, raising poultry, foraging, hunting, [or] fishing (Table 1). For more details, see the survey text in Supplemental materials.

### Data analysis

Quantitative data analysis was conducted using R Studio (version 2022.7.2 + 576 “Spotted Wakerobin”). R packages included tidyr, qualtRics, questionr, ggplot2, and viridis. Responses were retained for analysis if they answered questions regarding food security at the time of the survey and in the same period in 2019, and also answered questions regarding at least one wild or backyard food activity at the time of the survey and in the same period in 2019. Respondent characteristics were summarized using percentages for the total sample, and for each wild or backyard food activity sub-sample separately (respondents could be in multiple sub-samples).

First, food security, food assistance utilization, and use of traditional food sources were summarized for before and during the COVID pandemic, and McNemar’s tests performed to test whether percentages were equivalent for reports about 2019 and 2020 (Table 2). Second, we examined how production and consumption of wild/backyard foods shifted during the COVID-19 pandemic (Figure 1). The number of survey respondents who reported each pattern of change (“for the first time this year,” “much more this year,” “a little more this year,” “the same amount as last year,” “a little less this year, and “much less this year”) in production and consumption of each type of wild/backyard food (fruit & vegetables, eggs, poultry, foraged foods, fish or seafood, wild game) was determined. For the total sample and the sub-sample of respondents who reported wild/backyard food production of each food type (e.g., fish for fishers), we described consumption of relevant foods as a percentage of whoever consumed the item from each relevant source and the modal times per month consumed among those who ate it (Table 3). Third, we examined associations between challenges (food insecurity, job loss) or use of food assistance with perceived increase in production or consumption of any wild and backyard foods and tested with Fisher’s exact test analysis (Table 4) to accommodate the small sample size for poultry rearers and foragers. All tests were considered statistically significant at 95% confidence. For the Table 4 analyses, we combined those who reported producing or consuming a wild and backyard food “for the first time this year,” “much more this year,” or “a little more this year” production/consumption into “increased.”

**Table 2:** Perceived changes in food security, food assistance utilization, and food sources before and during the COVID-19 pandemic.

	2019 *n* (%)	Since the COVID-19 outbreak *n* (%)	*p*-value
**Participation in wild and backyard food production**			
Gardening	303 (60.0)	307 (60.8)	0.77
Backyard poultry	48 (9.5)	46 (9.1)	0.86
Foraging	108 (21.4)	121 (24.0)	0.086
Fishing	134 (26.5)	124 (24.6)	0.203
Hunting	142 (28.1)	143 (28.3)	1.0
**Food security**			
High or marginal food security	398 (93.4)	378 (87.7)	**<0.001**
Low food security	19 (4.5)	38 (8.8)	**<0.001**
Very low food security	9 (2.1)	15 (3.5)	**<0.001**
**Food sources**			
Grocery store	447 (96.8)	411 (89.0)	**<0.001**
Convenience or corner store	155 (33.5)	140 (30.3)	**0.041**
Specialty food store	181 (39.2)	149 (32.3)	**<0.001**
Grocery delivery (like Amazon or Instacart)	43 (9.3)	154 (33.3)	**<0.001**
Meal-kit delivery (like Home Chef)	32 (6.9)	32 (6.9)	1.0
Meals on Wheels	1 (0.2)	2 (0.4)	1.0
Restaurant to-go	274 (59.3)	370 (80.1)	**<0.001**
Restaurant eat-in	355 (76.8)	154 (33.3)	**<0.001**
Farmers’ market	307 (66.5)	218 (47.2)	**<0.001**
Direct from farm (CSA, farm stand pickup/delivery)	128 (27.7)	145 (31.4)	0.065
Other (Self-describe)	21 (4.5)	29 (6.1)	0.061
**Food assistance**			
SNAP or Food Stamps (including COVID-19-EBT or P-EBT)	24 (7.1)	38 (11.2)	**0.014**
WIC (Women, Infant, and Children’s Program)	11 (3.3)	8 (2.4)	0.51
Free or Reduced-price school meals	37 (10.9)	47 (13.9)	0.1003
Food pantry/Food bank	22 (6.5)	51 (15.1)	**<0.001**
Food or money for food from family, friends, or neighbors	14 (4.1)	46 (13.6)	**<0.001**
Food or money for food from a religious community	4 (1.2)	21 (6.2)	**<0.001**
Other food assistance program (e.g., Commodity Supplemental Food program, Meals on Wheels)	4 (1.2)	14 (4.1)	**0.0094**
None used	222 (65.7)	212 (62.7)	**0.024**
**Job disruption (*n* = 418)**			
**Have you or anyone in your household experienced a loss of income or job since the COVID-19 outbreak? (multiple responses across rows and columns possible)**	**Happened at all since COVID-19 *n* (%)**	**Still happening today *n* (%)**	
Yes, lost job	41 (9.8)	30 (7.1)	
Yes, reduced hours or income at job	58 (13.9)	54 (12.9)	
Yes, furloughed	35 (8.4)	9 (2.2)	
No, have not had any loss of job or income	274		

*p*-values in bold show a significant change in participation before and after the pandemic.

**Table 3:** Consumption of wild and backyard foods in total sample and among respondents producing relevant wild and backyard foods.

	All surveyed		Respondents producing wild and backyard foods (e.g., gardeners for fruit/vegetables)	
Over the past 3 months, how many times have you eaten [food] from the following sources?	Ever consumed (%)	Average consumption freq. (times/mo., among those consuming)	Ever consumed (%)	Average consumption frequency (times/mo., among those consuming)
**Fruit and vegetables**				
Fruit and vegetables from my garden	75.7	13.4	97.6	13.5
Fruit and vegetables grown by friends, family and neighbors	61.3	5.4	63.4	5.2
Fruits and vegetables purchased from a farm or farmers (including CSAs)	76.2	8.8	81.2	9.1
Fruits and vegetables purchased from a store	98.8	16.3	98.6	16.6
**Egg consumption**				
From poultry that I raised	12.4	16.5	92.7	16.4
From poultry raised by friends, family, and neighbors	23.3	7.2	19.5	12.0
Purchased from a farm or farmers’ market	31.0	9.2	14.6	6.7
Purchased in a store	83.4	10.3	34.1	5.1
**Poultry consumption**				
From poultry that I raised	6.5	6.7	51.2	5.8
From poultry raised by friends, family, and neighbors	8.6	3.2	17.1	2.6
Purchased from a farm or farmers’ market	17.8	3.8	9.8	6.0
Purchased in a store	90.7	7.7	78.0	5.3
**Foraged foods**				
Foods foraged by me	34.0	4.5	93.8	4.6
Foods foraged by friends, family or neighbors	14.8	3.1	30.4	3.4
Foraged foods purchased from a farm or farmers’ market	23.1	3.2	29.5	4.2
**Fish or seafood**				
Fish or seafood that I caught	23.4	3.1	69.7	3.1
Fish or seafood caught by friends, family or neighbors	16.7	2.3	31.1	2.2
Fish or seafood that was purchased	81.0	3.9	83.2	3.9
**Wild game**				
Wild game that I caught	28.1	4.8	80.1	4.8
Wild game caught by friends, family or neighbors	22.3	3.1	36.8	3.6
Wild game that was purchased	1.9	5.0	2.2	4.7

**Table 4:** Associations between pandemic-related challenges and increased wild and backyard food production.

	Food secure (%)	Food insecure (%)	*p*	No food assistance (%)	Any food assistance (%)	*p*	No job/income loss (%)	Job/income loss (%)	*p*
Gardening	36.1	52.8	**0.02**	33.8	50.8	**0.05**	34.7	45.8	0.23
Poultry raising	5.3	13.2	0.48	4.4	11.7	1.0	3.8	11.8	**0.04**
Foraging foods	11.2	17.0	0.19	9.6	19.2	0.07	11.0	13.9	0.37
Fishing	11.7	11.3	0.41	10.8	14.2	1.0	11.6	11.8	0.23
Hunting	12.8	13.2	1.0	12.4	14.2	0.41	12.7	13.2	0.10

*p*-values in bold show a significant association between pandemic-related challenges and production.

## Results

Sixty-five percent of respondents gardened, 9.3% raised poultry, 25.5% foraged, 27.6% fished, and 31.6% hunted/planned to hunt in 2020 (Table 1). A small majority of respondents were female (54%). A higher proportion of women than men gardened (65.7% female), reared poultry (70%), and foraged (58.2%), while fishers were nearly balanced between genders (50.4% male and 47.9% female), and the majority of hunters were men (55.9% male and 39.7% female). Survey respondents were highly educated relative to the wider Upstate New York community; a majority of respondents had completed associates, bachelor, or postgraduate degrees (68%). More than three-quarters of respondents were non-Hispanic white (77.5%). People who gardened were relatively more diverse than those who engaged in other activities, in terms of race, income, and education levels. Respondents were relatively high income with 47.3% making more than $75,000, and only 10.3% making less than $25,000. These racial, and gender demographics also differ from the United States 2015 census data of the counties surveyed. Specifically, our survey population skews more white and more female than the census, validating the expectation that the sample would not be representative of central/upstate New York (Table S1, Supplementary material). Instead, it captures a subset of wild and backyard food users who are linked with Cornell Cooperative Extension, New York Department of Environmental Conservation, social media pages aimed at these activities, and who have access to the internet.

About a third of respondents (34.4%) reported that the COVID-19 pandemic had negatively affected their income (Table 2). Compared to 2019, food insecurity significantly increased among survey respondents. Low food security more than doubled to 8.8% and very low food security rose by two-thirds to 3.5% (total food insecurity in 2020 was 12.3%). Respondents’ use of food assistance also increased, including significant increases in use of food banks and pantries; food and money from family, friends, neighbors, and religious communities; SNAP or WIC (including COVID-19-EBT or P-EBT); and other food assistance programs. Choices about conventional food sourcing also changed from 2019 to 2020. Grocery delivery and the use of restaurant to-go orders increased while use of grocery stores, specialty food stores, farmer’s markets, restaurant eat-in orders, and convenience stores decreased.

Across the wild and backyard food production strategies, 4.0–14.3% of respondents reported engaging for the first time and 39.6–45.7% reported increased production a little or a lot more (Figure 1A). 31.6–42.7% of respondents’ production was the same as the previous year. A notable minority of 8.3–21.5% of people, however, reported decreased production (either a little or a lot less) of wild and backyard foods. As there are relatively few people who participated in wild and backyard food use in only 2019 or 2020, there is no significant difference between participation across years (Table 2). More substantial shifts are observed, however, in the intensity of participation in while and backyard food production, and these shifts in engagement were remarkably similar across activities.

Changes in wild and backyard food consumption followed very similar patterns to production (Figure 1B). While few people consumed these foods for the first time, 23.9–55.6% increased their consumption a little or a lot more, with consumption of gardened fruits and vegetables growing most (55.6%). Across food types, 36.6–61.4% of respondents’ consumption was the same as the previous year. Again, a notable minority of 4.8–22.0% of people reported decreased consumption (a little or a lot less) of wild and backyard foods.

People producing wild and backyard foods were, as expected, much more likely to report they ever consumed the foods they produced (Table 3). Consumption of gardened fruit and vegetables (97.6%), eggs from backyard poultry (92.7%) and foraged foods (93.8%) was nearly ubiquitous among producers, while a majority also consumed poultry meat (51.2%), fish they caught (69.7%), and wild game they hunted (80.1%). Large proportions of respondents also reported that they consumed wild and backyard foods shared with them by friends, family, and neighbors. These proportions were slightly larger for producers of wild and backyard foods, underscoring these social networks, yet over 60% of all respondents had received shared fruit and vegetables and more than 20% had received eggs and wild game. Among those consuming these foods, consumption frequency was substantial. Average consumption of gardened fruit and vegetables (13.5 times/month) and eggs (16.4 times/month) were very high, while average consumption of poultry meat, foraged foods, fish, and wild game ranged from 3.1 to 5.8 times/month.

There was a significant association between food insecurity and increased gardening effort (*p* = 0.02), use of food assistance and increased gardening effort (*p* = 0.05), and job loss and increased poultry-rearing effort (*p* = 0.04; Table 4). Experiences of hardship (food insecurity, job loss, and food assistance use) were not associated with increased consumption of wild and backyard foods (Table S2, Supplementary material).

The number of respondents motivated to produce food through all strategies to “have more control over food availability” increased between 2019 and 2020 (*p* < 0.001 to <0.05; Table 5). Gardeners were also more likely to report the following motivations in 2020: “have more affordable ways of getting food” (*p* < 0.001), “get outside” (*p* < 0.001), “be active” (*p* < 0.001), “have more control over food quality” (*p* < 0.05), and “keep my kids occupied and learning” (*p* < 0.05). A larger sample size for gardeners (*n* = 357) may have facilitated detection of statistically significant differences in motivations. However, poultry rearers, foragers, fishers, and hunters also saw more people reporting the same motivations in 2020 as compared to 2019, suggesting a wider set of shifting motivations for other wild and backyard food producers as well.

**Table 5:** Drivers of production of wild and backyard foods from 2019 to 2020.

Motivations as drivers	Garden (*n* = 357)				Poultry (*n* = 62)				Forage (*n* = 139)				Fish (*n* = 154)				Hunt (*n* = 163)			
	2019	2020	% dif.	*p*	2019	2020	% dif.	*p*	2019	2020	% dif.	*p*	2019	2020	% dif.	*p*	2019	2020	% dif.	*p*
Have more control over food availability	44.0	59.9	16.0	***	40.3	69.4	29.0	***	55.4	67.6	12.2	*	49.4	62.3	13.0	**	50.3	59.5	9.2	*
Have more affordable ways of getting food	49.3	59.4	10.1	***	45.2	56.5	11.3	0.17	59.7	66.2	6.5	0.14	55.2	63.6	8.4	**0.055**	52.1	57.7	5.5	0.16
Get outside	71.4	80.1	8.7	***	64.5	66.1	1.6	1	79.1	83.5	4.3	0.31	76.0	79.2	3.2	0.46	77.3	77.3	0.0	1
Be active	66.4	73.1	6.7	***	58.1	64.5	6.5	0.39	71.9	77.0	5.0	0.25	70.8	74.7	3.9	0.39	74.2	74.8	0.6	1
Have more control over food quality	66.9	72.5	5.6	*	74.2	74.2	0.0	1	79.9	80.6	0.7	1	67.5	72.7	5.2	0.19	68.7	73.6	4.9	0.19
Keep my kids occupied and learning	19.6	23.2	3.6	*	40.3	45.2	4.8	0.55	27.3	32.4	5.0	0.07	25.3	30.5	5.2	0.12	24.5	27.6	3.1	0.27
Build relationships with people with shared interests in producing food	24.9	28.0	3.1	0.11	29.0	25.8	−3.2	0.68	35.3	36.0	0.7	1	29.9	30.5	0.6	1	28.8	28.8	0.0	1
Do something good for the environment	56.3	58.3	2.0	0.36	45.2	45.2	0.0	1	63.3	63.3	0.0	1	53.2	55.2	1.9	0.68	54.6	54.0	−0.6	1
Participate in a cultural tradition	24.9	24.9	0.0	1	29.0	22.6	−6.5	0.29	34.5	33.1	−1.4	0.75	30.5	29.2	−1.3	0.81	31.9	30.7	−1.2	0.81
Other	5.6	5.3	−0.3	1	9.7	8.1	−1.6	1	8.6	7.2	−1.4	0.68	6.5	5.8	−0.6	1	5.5	6.7	1.2	0.62

Non-significant *p*-values are printed in normal text. Suggestive p-values are in bold text. Significant p-values are marked with *p ≤ 0.05; **p ≤ 0.01; ***p ≤ 0.001.

## Discussion

During the COVID-19 pandemic, this limited sample of people in upstate New York remade their own food environments by increasing production of wild and backyard foods. This sample of more highly educated, whiter, and relatively wealthy New Yorkers had lower rates of food insecurity than other United States samples [see Niles et al. (6) for comparisons]. Nevertheless, these findings show our sample was motivated to intensify production and consumption of wild and backyard foods to increase the control they had over their food environment during a time of widespread uncertainty.

Across production activities, 39.6–45.7% of respondents increased production (either a little or a lot more) and 4.0–14.3% produced wild and backyard foods for the first time. Further, 23.9–55.6% increased their consumption of the foods they produced. More than half of respondents reported they were motivated to choose home production to control food availability, a key part of food security. Amid increased uncertainty in the conventional food environment, production of wild and backyard foods allowed individuals to exert control over their food environments and particularly to improve access to and control of nutritious, perishable foods.

Recalling the acute impact of the COVID-19 pandemic’s outbreak phase is vital to understanding our findings. In the first six-ten months (between pandemic onset in February–March 2020 and the survey in October–December 2020) conventional supply chains faltered nationally and globally, grocery store shelves emptied, and communities faced widespread uncertainty about their food security. Conventional meat production supply chains in the United States were particularly hard hit in 2020 as meat packing facilities struggled with COVID outbreaks and stock outs were common (56). Fresh fruit and vegetable markets initially wobbled, before adapting over time (57). Meanwhile, income loss was common: 34.4% of this study’s respondents and 43.5% of respondents in a multi-site United States study faced income loss (6). Between supply chain shifts and income loss, food insecurity rose. In this study sample food insecurity (combined low and very low food security) doubled from 6.6 to 12.3% between 2019 and 2020 (Table 2). A smaller proportion of our respondents was food insecure compared to findings from across the state and nation, which in 2020 ranged from 30.2 to 54.3%. The higher values were observed in communities that were high risk and/or included large numbers of black, indigenous, and other people of color (6).

### Adapting to a crisis food environment through shifts in production

Within this larger context and this study’s specific sample, gardening production and consumption in upstate New York particularly expanded (of gardeners, 56.6% increased or started new production, 56.3% increased or started new consumption). Most, however, self-reported not as new gardeners, but as increasing compared to previous effort. Those who were “new” may also have been returning to gardening after a period of inactivity. This may represent an intensified application of local knowledge and practices by local communities [as defined by the ICCA Consortium in (58)] that are part of the culture (broadly construed) of Upstate New York. It may also represent activation of local knowledge and practices that respondents already had but were not using.

The expansion of gardening is consistent with other data showing increased gardening activity in response to the COVID-19 pandemic in the Global North (15, 17, 18). Corresponding to this increased production and consumption, respondents reported moderate decreases in shopping at farmers’ markets over the same period (−19.3% pts). Farmers’ markets in the United States reported closures or large decreases in sales during COVID-19 lockdowns (59) but our novel data suggest that increases in gardening also may have contributed to reductions in farmers’ market shopping.

Increased gardening was associated with food insecurity, while a reliance on food assistance and experiencing job loss were associated with poultry rearing (Table 4). Though we cannot ascertain the directionality of these relationships, the experience of hardship may have motivated increased gardening or poultry effort or allowed more time for these activities. Even as gardening and poultry rearing may be adaptive strategies, additional support from local and regional organizations for these households may be needed.

However, the impact of COVID-19 on wild and backyard food production was not a monolith even within our sample. While most individuals in this study maintained or increased production effort, a notable minority decreased efforts instead. Gardening, poultry rearing, foraging, and hunting saw decreases in 8.3–10.8% of respondents, and 21.5% of fishers decreased their efforts. Time is often a key constraint to fish and wild game harvest, even for people who value it for deeply held reasons (60). While “essential” workers and parents may have had decreased time available, “non-essential” workers who stayed home during 2020’s lockdown may have had increased free time, contributing to the variability in our responses. Seasonality may also have influenced these trends. For gardeners planting begins early in the year, which was at the height of pandemic’s supply chain bottlenecks, store closures, and reluctance to enter public spaces. Respondents may therefore have been especially motivated to adopt gardening in just the right part of the season for gardening to begin. Likewise, certain hunting and fishing seasons (e.g., turkey season) begin early (April and May) and may have similarly shifted in the immediate aftermath of the pandemic declaration (13, 39). Thus for respondents who were able to continue or increase home production, wild and backyard foods often contributed to diets.

### Wild and backyard foods were widely consumed

Backyard food producers (gardeners and backyard poultry rearers) in this sample almost universally consumed the often nutritious and perishable foods they produced. Nearly all gardeners ate fruits and vegetables they grew, with an average frequency of 13.5 times/month. Assuming consumption of average portion sizes, that average frequency provides 17% of the minimum recommended monthly fruit and vegetable consumption [based on dietary guidelines from the (61)]. Our findings are in line with previous studies, which found higher fruit and vegetable intake from any source among other home food producers during the COVID-19 pandemic (52). Similarly, almost all poultry-rearers in our sample (93%) ate the eggs they produced and half (52.3%) ate home-reared meat. The average frequency of consumption for these was high, 16.4 times/month for home-produced eggs and 5.8 times/month for home-produced poultry meat. The US Department of Agriculture (USDA) considers both eggs and poultry as protein foods so, assuming average portion sizes, together this reported average frequency of consumption fulfills approximately 53% of the USDA’s monthly recommendation for protein foods (61). Given concerns that diet quality worsened during the early pandemic (62), the contribution of gardening and poultry rearing to meet needs for protein food and fruits and vegetables may have buffered or even reversed negative food environment shifts in access to these foods in upstate New York and similar North American contexts.

Wild foods, or foraged, fished, and hunted foods, were also consumed by strong majorities of producers in this sample. Nearly all foragers (94%), 70% of fishers, and 80% of hunters consumed their own wild foods, though consumption frequencies were relatively lower (4.6 times/month for foraged foods, 3.1 times/month fish; 5.8 times/month wild game). The lower average frequencies of wild foods consumption may be related to the higher investments needed to undertake these activities (e.g., licensing, travel to appropriate sites, etc.) or higher variability of success, compared to gardening and poultry-rearing. Accessing wild foods is qualitatively different from producing backyard foods due to the need to travel to suitable habitats, find and harvest the target species, as well as high levels of safety knowledge required (i.e., species identification, gun safety, and contaminant-related consumption guidelines). It also depends upon some level of chance for actually encountering the desired species.

We also find indications that people in our sample who produced wild and backyard foods were part of a network that affords additional access to wild and backyard foods. Beyond consuming their own foods, about twice as many foragers, fishers, and hunters consumed foraged foods, fish, and wild game harvested from friends, family, or neighbors in comparison to the sample overall. Twice as many poultry rearers ate poultry from food sharing than the sample overall. This suggests that increased access to food sharing also benefits the diets of these home producers. Although we cannot ascertain if this is an increase in sharing, this finding is consistent with other studies from around the world that found an increase in food sharing, trading, or bartering during the COVID-19 pandemic, sometimes as an extension of traditional kin networks and sometimes in broader and even digital communities (63–65). Fish sharing within small-scale fisheries worldwide during the COVID-19 pandemic has also been reported (63, 64) though less is known about sharing of other wild and backyard foods, a gap that our study helps to fill for places similar to our study area.

### Control over food availability and affordability motivates food environment shifts

Strong majorities of participants in our sample who produced wild and backyard foods consistently reported they were driven by two types of motivations, (1) to get outside and be active and (2) control food quality, availability, and affordability. However, only one motivation identified by respondents had a statistically significant increase between 2019 and 2020 for gardening, poultry rearing, foraging, fishing, and hunting: have more control over food availability. This seems likely in response to the massive uncertainty the pandemic engendered in food and social systems and illustrates how these households perceived their use of wild and backyard foods at this time. Several other motivations to garden also increased significantly between 2019 and 2020 including food affordability, control over food quality, and keeping active and outdoors. While poultry rearers, forager, fishers, and hunters increased their identification of these as motivating, the increases were not significant. Given that these production methods had a 2–3 times smaller sample size than gardening, we interpret that lack of significance as less convincing as we would with equal sample sizes. Despite the smaller sample size, affordability as a motive for fishing still had a suggestive value of p (*p* = 0.055), which we interpret as showing the importance of fishing for procuring an affordable protein food. Taken, together, these findings are consistent with prior research identifying food access and cost savings as motivators for COVID gardening across the Global North (20), and provides novel evidence that pandemic-era motivations for poultry-raising, foraging, fishing, and hunting were similar. It is also consistent with recent literature theorizing more broadly about the potential role for wild food provisioning as a support for food security worldwide during crises such as COVID-19 (66, 67), and the role of local knowledge and practices as a buffer and cultural strength recently advocated in human ecology literature (68).

If respondents perceived their use of wild and backyard foods as improving control over food availability and access, or allowing them to practice stress-reducing hobbies, these activities may have been key to reducing pandemic-related stress. Around three-quarters of respondents selected the motivations of “get[ting] outside” and “be[ing] active” consistently between 2019 and 2020. While there was no statistically significant change in identification of these motivations, the high proportion of respondents choosing them may indicate that these behaviors were providing stress reduction. This would be consistent with the widespread literature describing the ways that gardening mitigated pandemic-related stress (19–21, 26, 30–33), and are also consistent with stress reduction associated with pandemic fishing in Europe (46). A study in Vermont found higher stress in hunters and fishers than in gardeners (69). Given demographic differences between hunters and fishers compared to gardeners in our sample, we speculate differences in stress could be related to underlying factors, such as finances or social identity, as opposed to differences in how fishing, hunting, and gardening activities benefit individuals. We found higher proportions of men (20–25% more male identifying fishers and hunters than gardeners), and some studies have found higher levels of untreated stress and mental health problems in men than in women (70).

### Shifts seen during the COVID-19 crisis indicate need for ongoing community support

Regional service providers can use our findings to shed light on how wild and backyard food production and consumption intersected with shifting New York food environments. Our research indicates some areas that could be targeted to better support communities as they grapple with the long term fall out of COVID-19, some areas that similar future crises are likely to impact, and some areas that need further clarification. Additionally, it appears that there may be local knowledge and practices active in the communities respondents are part of, which could be linked to future management strategies. This application of local knowledge and practices in a time of crisis would be supportive of calls in the literature for better understanding, respect, use, and integration of local and traditional ecological knowledge (68).

In New York State, the New York Department of Environmental Conservation, Cornell Cooperative Extension offices, and food banks (among others) were forced to create COVID-19 relevant programming on the fly, while dealing with many of the same challenges as the public. For example, to help bridge knowledge barriers to gardening extension programs across the United States often link older, experienced gardeners (e.g., Cornell Cooperative Extension Master Gardeners) with newer gardeners, but the in-person components of these were canceled by New York On Pause. This forced programs to offer remote or online options with very little notice for development or implementation (71). Meanwhile, social distancing reduced space at popular fishing sites (72) and time and access at community gardens (71). Additionally, stock-outs of key materials due to COVID-related supply chain disruptions likely impacted adoption of wild and backyard food production (e.g., seed shortages in early 2020 could have delayed planting or increased costs). Despite these increased barriers, this study found evidence of increases in production effort concurrent with motivations shifting towards improving control over availability and access of wild and backyard foods. This validates the efforts made by service providers like Cornell Cooperative Extension and New York Department of Environmental Conservation and offers encouragement for robust planning to better meet community needs during future crises.

Increases in wild food use in particular also have environmental sustainability implications both positive and negative (73–76). Having more foragers, hunters, and fishers involved or increasingly involved could help address recruitment concerns for hunting and fishing participation, often a key goal of state agencies who rely on these groups to fund state programming for environmental stewardship (77, 78). Yet additional people means additional harvest pressure and more interactions between humans and wildlife. This requires conservation and resource managers to work closely with hunters, fishers, and foragers to facilitate meaningful access while still avoiding negative impacts of heavier use on state or local sustainability and conservation goals. Balancing harvest pressure management with positive outcomes of wild food use has also been a concern in global settings (73, 74). Wild flora can be particularly sensitive to such high harvest pressure. Foraging is also generally outside the purview of community and even regional level programming, perhaps due to the high-level knowledge necessary to safely identify wild mushrooms, herbs, vegetables, and fruits. This means that there are fewer options for building onto to manage higher foraging harvest pressures. These issues are akin to the challenges and solutions explored in discussions of traditional ecological knowledge and environmental management, which attempt to harmonize environmental governance with traditional hunting, fishing, and foraging (68).

Finally, the high levels of wild and backyard food consumption found in this research highlights the importance of education on food safety. The role that agencies like the New York Health Department of Health and the New York Department of Environmental Conservation play in communication of local contamination levels and consumption guidelines is vital, as is biosafety and food safety programing from Cornell Cooperative Extension. While the potential for high nutritional value food access is a clear benefit of wild and backyard food consumption, it must be balanced with risks around food safety and contaminant exposure. Achieving food safety while producing wild and backyard foods requires specific knowledge and skills, identification, potential for zoonotic disease transmission, safe food processing, preparation, and preservation. Expanding educational efforts in handling and preserving backyard foods (i.e., fruits, veggies, poultry, and eggs) to include food safety in wild food production may be appropriate in the study communities, or others like them [e.g., as in Seneca County Cornell Cooperative Extension’s Wild Table programming (79)]. This is all the more critical in a world that has experienced the disruption and tragedy a novel zoonotic infection causes, leading to intensified debate on the consumption of wild foods (e.g., 75, 80).

In addition to food safety, the contamination of soil, water, fish, and game can impact all wild and backyard foods. For example, lead contamination of backyard chicken eggs in (81), urban garden soils (82), and wild game contaminated by “forever chemicals” (83) or lead ammunition (84) are all potential concerns. People eating wild and backyard foods need to be able to contextualize the contamination risks of their whole diet (i.e., how many wild caught fish can be safely eaten if store bought fish, are also being consumed), without being overly intimidated. Concern about risks can reduce levels of consumption below what would provide health benefits, such as the case when pregnant women avoid fish that would be nutritionally beneficial (85–87).

Additionally, any language-based challenges in appropriately interpreting guidelines written in English may compound the difficulty of accurately contextualizing personal risk levels. To effectively strike the delicate balance of writing and sharing guidelines or offering programming that protects diverse communities successfully without discouraging individuals from benefiting from highly nutritious food requires in depth understanding of food environments, cultural practices, local risks. For example, one Western New York organization offers their local fishers state level fish consumption guidelines translated into five locally common languages (88). Robust and consistent future funding can support community outreach to ensure that the necessary knowledge and skills are in fact being used in specific New York communities who practice higher levels of wild and/or backyard food consumption (89).

### Study limitations

Our study used an online, convenience sample from upstate New York and our findings cannot be generalized as the sample was not representative of the population. In particular, respondents were disproportionately educated, higher income, and skewed towards non-Hispanic white individuals and women compared to the general population within the counties surveyed. These demographic biases limit this study’s ability to assess the impact of cultural and racial diversity, lower incomes, and lower levels of food security on production, consumption, and motivation findings, which is critical to fostering supportive services and systems for all wild and backyard food producers. Of note, global settings where engagement with wild and backyard foods may differ substantially, for example in settings where fishing is more often an occupation (90–92) experienced very different dynamics in response to COVID-19.

As we sought out individuals engaged in wild and backyard food production, we cannot estimate population level participation in these activities more widely from this data. Our survey was conducted in October–December 2020 and captured the early phase of the COVID-19 pandemic. Though we made efforts to minimize recall bias, respondents compared periods before and since the pandemic, likely introducing some recall bias. Because of the seasonality associated with wild and backyard food production, the survey timing also generated different recall periods for different activities (e.g., spring fishing, compared to fall garden harvests). Further, the majority of the first COVID-19-era deer hunting season occurred after our survey was conducted so that activity was reported prospectively. Seasonality of these differing production methods complicates comparisons across seasons. Finally, we did not directly ask about stress reduction as a stand-alone motivation, and so our analysis of the impact of these activities on stress is therefore limited. The timing of the data presented here (collected in 2020) did not provide evidence on whether pandemic-associated behavior change will be sustained in the long term or abandoned.

## Conclusion

While COVID-19 presented an acute shock to the food system, it also has had a long shadow. Supply chains have struggled to return to pre-COVID-19 functionality, food prices remain high (93) and have in some cases spiked even higher at times [e.g., egg prices in 2022 in the United States, (94)]. Although COVID-19 restrictions worldwide have largely been removed, the socio-economic impacts of COVID-19 remain widespread and new crises from extreme weather to political upheaval may acutely threaten food systems in the future. Understanding the extent to which and why individuals included wild and backyard food production as part of their food environments will be valuable for planning for and mitigating future crises. Organizations such as New York Department of Environmental Conservation, Cornell Cooperative Extension, and others can use our findings to tailor their current and future support for wild and backyard food production and investigate whether more vulnerable communities are also benefiting as the studied communities did here. For example, the significant relationship between increased gardening effort and food insecurity points to a potential opportunity to further support food insecure households through gardening specific programs. During or after a crisis, wild and backyard food production may support physical and mental health through nutrition, stress relief, and exercise. Producers of these foods have also long been key supporters of environmental sustainability within the woods, waterways, and lands that they use, enabling an even broader contributions their food environments. In a modern world grappling with sustainability, climate change, and socio-political challenges, wild and backyard food production empowers households by letting them exert control over their own food environments and adapt to challenges.

## Data availability statement

The raw data supporting the conclusions of this article will be made available by the authors, without undue reservation.

## Ethics statement

This study involved humans and was exempted from IRB review by the Cornell Institutional Review Board (Protocol ID#: 2008009765). The study was conducted in accordance with the local legislation and institutional requirements. The participants provided their written informed consent to participate in this study. Written informed consent was obtained from the individuals for the publication of any potentially identifiable data included in this article.

## Author contributions

JC-S: study design, funding, data collection, early data analysis and synthesis, and writing. NC: data analysis on food security. GM: survey design and administration. LL: early analysis of backyard poultry data. AT: early analysis of hunting and foraging data. ZW: early analysis of fishing data. SY: early analysis of gardening data. AS and KH: study design, analysis, and revisions. KF: advising, study design, coordination, analysis supervision, and writing. All authors contributed to the article and approved the submitted version.

## Conflict of interest

The authors declare that the research was conducted in the absence of any commercial or financial relationships that could be construed as a potential conflict of interest.

## Publisher’s note

All claims expressed in this article are solely those of the authors and do not necessarily represent those of their affiliated organizations, or those of the publisher, the editors and the reviewers. Any product that may be evaluated in this article, or claim that may be made by its manufacturer, is not guaranteed or endorsed by the publisher.

## Footnotes

^1^Syracuse.com
