# Supplementary material for: Wild and backyard food use during COVID-19 in upstate New York, United States
Source: Front Nutr. 2023 Sep 5;10:1222610. doi: 10.3389/fnut.2023.1222610 (PMC10507697; doi:10.3389/fnut.2023.1222610)
Supplement: Supplementary file 1 [file Data_Sheet_1.PDF]

NY COVID19 Food Sourcing

Note that this survey questionnaire text is presented without skip logic for simplicity.

S1 In which county do you reside?

- ○ Broome
- ○ Cayuga
- ○ Cortland
- ○ Onondaga
- ○ Oswego
- ○ Seneca
- ○ Not one of the above

Page Break

*

S2 In what year were you born?

____

End of Block: Screener

Start of Block: Eligible

Elig

Thank you for your interest in this survey and for taking the time to answer these initial questions. Please click 'continue' below to complete the main survey.

End of Block: Eligible

Start of Block: Ineligible

Inelig Thank you for your interest in this survey and for taking the time to answer these initial questions. Based on your responses you are not eligible for the survey. Please contact Jeanne Coffin-Schmitt at jlc558@cornell.edu if you have any questions.

End of Block: Ineligible

Start of Block: Intro

Intro In this survey we will refer to the time before the COVID-19 outbreak as "2019". For the time after the COVID-19 outbreak, please think about the time period since March 2020.

Definitions for some words can be seen if you hover over or tap the word.
When we ask about "foraging" in this survey, foraging means searching for and harvesting wild produce, including berries or other fruits, greens or other vegetables, roots, mushrooms, and medicinal plants.
When we ask about "CSAs" in this survey, we mean a partnership called "community supported agriculture" between a local farm and a customer with a set price for a ‘share’ of the farm’s harvest and delivery or pick-up options.

End of Block: Intro

Start of Block: Food Procurement General

FPG_text In this section we will ask you about how you usually get food.

Page Break

1 Did you or anyone in your household do any of the following to get food? Please answer for both time periods. Check all that apply.

	2019	Since the COVID-19 outbreak
Planted a garden	☐	☐
Raised chickens/poultry	☐	☐
Went foraging	☐	☐
Went hunting or intend to hunt later in 2020	☐	☐
Went fishing	☐	☐
None of the above	☐	☐

Page Break

FPG_text The following five questions ask whether anyone in your household wanted to get wild or backyard food but was unable to do so.

2 Did anyone in your household want to **garden**, but could not? Check all that apply. If neither apply, leave blank.

- ▢ In 2019
- ▢ Since the COVID-19 outbreak

3 Did anyone in your household want to **raise chickens or poultry**, but could not? Check all that apply. If neither apply, leave blank.

- ▢ In 2019
- ▢ Since the COVID-19 outbreak

4 Did anyone in your household want to **forage**, but could not? Check all that apply. If neither apply, leave blank.

- ▢ In 2019
- ▢ Since the COVID-19 outbreak

5 Did anyone in your household want to **hunt**, but could not? Check all that apply. If neither apply, leave blank.

- [ ] In 2019
- [ ] Since the COVID-19 outbreak

---

6 Did anyone in your household want to **fish**, but could not? Check all that apply. If neither apply, leave blank.

- [ ] In 2019
- [ ] Since the COVID-19 outbreak

Page Break

7a Which of the following did you or your household use to get food? Please answer for both time periods. Check all that apply.

	2019	Since the COVID-19 outbreak
Store: **Grocery store**	☐	☐
**Convenience or corner store**	☐	☐
**Specialty food store**	☐	☐
Delivery: **Grocery** delivery (like Amazon or Instacart)	☐	☐
**Meal-kit** delivery (like “Home Chef”)	☐	☐
**Meals on Wheels**	☐	☐
Restaurant: **To go**	☐	☐
**Eat in**	☐	☐
Alternative/Local: **Farmers' market**	☐	☐
**Direct from farm** (CSA, farm stand pickup/delivery)	☐	☐
**Other (please specify below if selected)**	☐	☐

7b Other before COVID, in 2019:

____

7c Other after COVID, since March 2020:

____

Page Break

8a Which of the following food assistance programs did you or your household use? Please answer for both time periods. Check all that apply.

	2019	Since the COVID-19 outbreak
**SNAP or Food Stamps** (including COVID 19-EBT or P-EBT)	☐	☐
**WIC** (Women, Infant and Children's Program)	☐	☐
**Free or Reduced-price School Meals** (lunch, breakfast, or summer meals)	☐	☐
**Food pantry/ Food bank**	☐	☐
**Food or money for food from family, friends, or neighbors**	☐	☐
**Food or money for food from a religious community** (church, mosque, temple, etc.)	☐	☐
**Other food assistance program** (Commodity Supplemental Food program, Meals on Wheels, or other)	☐	☐
None used	☐	☐

8b Please list other food assistance programs used **during 2019**:

____

8c Please list other food assistance programs used **since** the COVID-19 outbreak:

____

End of Block: Food Procurement General

Start of Block: Motivation for Choosing Food Procurement Methods

9a For foods that you (or your household) have or will **produce or harvest yourself** (through gardening, raising poultry, foraging, hunting and fishing), **why did you choose to get food that way?** Please answer for both time periods. Select all that apply. **I wanted to...**

	**In 2019**	**Since the COVID-19 outbreak**
Have more control over food quality	☐	☐
Have more control over food availability	☐	☐
Have more affordable ways of getting food	☐	☐
Be active	☐	☐
Get outside	☐	☐
Keep my kids occupied and learning	☐	☐
Participate in a cultural tradition	☐	☐
Build relationships with people with shared interests in producing food	☐	☐
Do something good for the environment	☐	☐
Other	☐	☐

9b Other - please describe:

____

Page Break

10a For foods that you (or your household) got from **local sources** like farm stands, farmers markets, and/or CSAs, **why did you choose to get food that way?** Please answer for both last year and since the COVID-19 outbreak (since mid-March). Select all that apply. **I wanted to...**

	In 2019	Since the COVID-19 outbreak
Have more control over food quality	▢	▢
Have more control over food availability	▢	▢
Have more affordable ways of getting food	▢	▢
Help support local farms and businesses	▢	▢
Build relationships with people with shared interests in local food	▢	▢
Do something good for the environment	▢	▢
Other	▢	▢

10b Other - please describe:

____

End of Block: Motivation for Choosing Food Procurement Methods

Start of Block: Gardening

Gardening_text You indicated that your household planted a garden. In this section we will ask about your gardening practices.

Page Break

11 Which of the following best describes your gardening this year as compared to last year? **I gardened:**

- ○ for the **first time** this year
- ○ **much more** this year
- ○ **a little more** this year
- ○ **the same** amount as last year
- ○ **a little less** this year
- ○ **much less** this year

Page Break

12a Where did/do you have your garden? Check all that apply. **I garden...**

	In 2019	Since the COVID-19 outbreak
At home in my yard	▢	▢
At home on my deck/window pots/fire escape	▢	▢
At a community garden	▢	▢
Other	▢	▢

12b Other - please describe:

____

Page Break

13 How big was/is your garden?   Estimate how many square feet your garden is:

Examples:

large round planter = 1 square foot
3' x 5' raised bed = 15 square feet
5' x 7.5' plot = 37.5 square feet

- ○ In 2019(# of square feet) ____
- ○ Since the COVID-19 outbreak   (# of square feet) ____

Page Break

14 What have you done with the foods you have harvested, or will harvest, from your garden this year? Please check all that apply.

	Eat right away	Preserve (dry, can, freeze)	Trade/give to others
**Vegetables**	☐	☐	☐
**Fruits**	☐	☐	☐
**Herbs**	☐	☐	☐

15 How do you think COVID-19 has changed your consumption of foods from your garden compared to last year? **I consumed foods from my garden:**

○ for the **first time** this year

○ **much more** this year

○ **a little more** this year

○ **the same**

○ **a little less** this year

○ **much less** this year

Page Break

16a How did you get skills and knowledge for your garden? Check all that apply.

	2019	Since the COVID-19 outbreak
I already had skills and/or knowledge	▢	▢
From family/friends	▢	▢
From online resources or courses	▢	▢
From an in-person course or workshop	▢	▢
From local gardening clubs or groups	▢	▢
Other	▢	▢

16b Other - please describe:

____

16c Please specify online resources/courses and in-person courses used:

____

Page Break

17a Has anything prevented you from gardening as much as you wanted to?

○ Yes

○ No

17b **What prevented you from gardening as much as you wanted to?**

	Not a barrier	Minor barrier	Moderate barrier	Major barrier
Lack space to plant a garden	○	○	○	○
Concerns about soil contamination	○	○	○	○
Lack information about how to garden	○	○	○	○
Lack skills required to use garden produce	○	○	○	○
Lack people to learn from	○	○	○	○
Cost of seeds, starts, soil, fertilizer, etc.	○	○	○	○
Time required to keep up with a garden	○	○	○	○
Other	○	○	○	○

17c Other - please describe:

____

End of Block: Gardening

Start of Block: Backyard Poultry

Poultry_text You indicated that you or your household has raised poultry (like chickens or ducks). In this section we will ask about your poultry raising practices.

Page Break

18 Which of the following best describes how many poultry birds (like chickens or ducks) you raised this year as compared to last year? **I raised:**

- ○ raised poultry for the **first time** this year
- ○ **many more** birds this year
- ○ **a few more** birds this year
- ○ **the same** number of birds as last year
- ○ **a few less** birds this year
- ○ **many fewer** birds this year

19a Where did/do you keep your poultry? Please answer for both time periods. Check all that apply. **I raised poultry...**

	In 2019	Since the COVID-19 outbreak
At home in my yard or farm	☐	☐
At a community garden or poultry hutch	☐	☐
Other	☐	☐

19b Other - please describe:

____

Page Break

20 How many poultry birds (like chickens or ducks) do you have?
Enter the number of birds you had/have:

- ○ In 2019(# of birds) ____
- ○ Since the COVID-19 outbreak(# of birds) ____

Page Break

21 What did you do with the food you have gotten, or will get, from your poultry this year? Please check all that apply.

	Eat right away	Preserve (freeze or pickle)	Trade/give to others
**Eggs**	☐	☐	☐
**Meat**	☐	☐	☐

22 How do you think COVID-19 has changed your consumption of eggs from your poultry compared to last year?
I consumed eggs from my poultry:

- ○ for the **first time** this year
- ○ **much more** this year
- ○ **a little more** this year
- ○ **the same**
- ○ **a little less** this year
- ○ **much less** this year

23 How do you think COVID-19 has changed your consumption of meat from your poultry compared to last year?
I consumed meat from my poultry:

- ○ for the **first time** this year
- ○ **much more** this year
- ○ **a little more** this year
- ○ **the same**
- ○ **a little less** this year
- ○ **much less** this year

Page Break

24a How did you get skills and knowledge for raising poultry? Check all that apply.

	In 2019	Since the COVID-19 outbreak
I already had skills and/or knowledge	☐	☐
From family/friends	☐	☐
From online resources or courses	☐	☐
From an in-person course or workshop	☐	☐
From local gardening clubs or groups	☐	☐
Other	☐	☐

24b Other - please describe:

____

24c Please specify online resources/courses and in-person courses used:

____

Page Break

25a Has anything prevented you from raising poultry as much as you wanted to?

- ○ Yes
- ○ No

25b **What prevented you from raising poultry as much as you wanted to?**

	Not a barrier	Minor barrier	Moderate barrier	Major barrier
Lack of land/space	○	○	○	○
Cost of feed, chicks, housing materials, etc.	○	○	○	○
Lack information about how to care for poultry	○	○	○	○
Lack skills to slaughter birds for meat	○	○	○	○
Lack skills for cooking eggs or meat from poultry	○	○	○	○
Lack people to learn from	○	○	○	○
Time required to care for poultry	○	○	○	○
Concerns about soil contamination	○	○	○	○
Other	○	○	○	○

25c Other - please describe:

____

End of Block: Backyard Poultry

Start of Block: Foraging

Foraging_text You indicated that you or your household forages or has foraged. In this section we will ask about your foraging practices.

When we ask about "foraging" in this survey, foraging means searching for and harvesting wild produce, including berries or other fruits, greens or other vegetables, roots, mushrooms, and medicinal plants.

Page Break

26 Which of the following best describes your <u>foraging</u> this year as compared to last year? **I <u>foraged</u>:**

- ○ for the **first time** this year
- ○ **much more** this year
- ○ **a little more** this year
- ○ **the same** as last year
- ○ **a little less** this year
- ○ **much less** this year

Page Break

27 **During the month you were foraging most**, about how many times did you go foraging?

○ In 2019(# of times per month)

____

○ Since the COVID-19 outbreak(# of times per month)

____

Page Break

28 What did you do with the foods you have already foraged, or will forage, this year? Please check all that apply.

	Eat/use right away	Preserve (dry, can, freeze)	Trade/give to others
**Wild vegetables**	☐	☐	☐
**Wild fruits**	☐	☐	☐
**Wild herbs**	☐	☐	☐
**Wild mushrooms**	☐	☐	☐
**Wild medicinal plants**	☐	☐	☐

29 How do you think COVID-19 has changed your consumption of foraged foods compared to last year? **I consumed foods I foraged:**

- ○ for the **first time** this year
- ○ **much more** this year
- ○ **a little more** this year
- ○ **the same** as last year
- ○ **a little less** this year
- ○ **much less** this year

Page Break

30a Where did you/do you plan to forage? Check all that apply. **I forage on...**

	In 2019	Since the COVID-19 outbreak
Public land (i.e. state forest, community space, etc.)	☐	☐
Land I own	☐	☐
Privately owned land I use for free	☐	☐
Privately owned land I pay to use	☐	☐
Other	☐	☐

30b Other - please describe:

____

Page Break

31a How did you get skills and knowledge to forage?   Check all that apply.

	In 2019	Since the COVID-19 outbreak
I already had skills and/or knowledge	☐	☐
From family/friends	☐	☐
From online resources or courses	☐	☐
From an in-person course or workshop	☐	☐
From local gardening clubs or groups	☐	☐
Other	☐	☐

31b Other - please describe:

____

31c Please specify online resources/courses and in-person courses used:

____

Page Break

32a Has anything prevented you from <u>foraging</u> as much as you wanted to?

- ◯ Yes
- ◯ No

32b **What prevented you from <u>foraging</u> as much as you wanted to?**

	Not a barrier	Minor barrier	Moderate barrier	Major barrier
Lack of access to land to forage on	○	○	○	○
Lack information about how to find wild plants	○	○	○	○
Lack skills required to preserve foraged produce	○	○	○	○
Lack people to learn from	○	○	○	○
Time or distance required to go foraging	○	○	○	○
Cost of courses on foraging	○	○	○	○
Concerns about wild plant or mushroom identification and poisoning	○	○	○	○
Concerns about soil contamination where wild plants grow	○	○	○	○
Other	○	○	○	○

32c Other - please describe:

____

End of Block: Foraging

Start of Block: Hunting and Wild Game

Hunting_text You indicated that you or your household hunted or plans to hunt. In this section we will ask about your hunting practices.

Page Break

33 Which of the following best describes your hunting or hunting plans for this year compared to last year?
I hunted/will hunt:

- ○ for the **first time** this year
- ○ **much more** this year
- ○ **a little more** this year
- ○ **the same** as last year
- ○ **a little less** this year
- ○ **much less** this year

34 Did you hunt in New York State (NYS) last year?

- ○ Yes
- ○ No

Page Break

35a What animals have you already hunted, or will you hunt? Please check all that apply.

	In 2019	Since the COVID-19 outbreak
White tailed deer	▢	▢
Waterfowl	▢	▢
Turkey	▢	▢
Upland birds (e.g. grouse, pheasant)	▢	▢
Small game	▢	▢
Other	▢	▢

35b Other - please describe:

____

Page Break

36 How do you think COVID-19 will change your consumption of wild meat that you hunted compared to last year?
I will or have consumed wild meat that I hunted:

- ○ for the **first time** this year
- ○ **much more** this year
- ○ **a little more** this year
- ○ **the same**
- ○ **a little less** this year
- ○ **much less** this year

Page Break

37a Where did you/do you plan to hunt? Check all that apply. **I hunt on...**

	In 2019	Since the COVID-19 outbreak
Public land (i.e. state forest, national forests)	▢	▢
Land I own	▢	▢
Privately owned land or access point I use for free	▢	▢
Privately owned land or access point I pay to use	▢	▢
Other	▢	▢

37b Other - please describe:

____

Page Break

38a How did/will you get skills and knowledge for hunting?
(Skills and knowledge include tactics, gun safety, meat processing and preparing, etc.)
Check all that apply.

	In 2019	Since the COVID-19 outbreak
I already had skills and/or knowledge	☐	☐
From family/friends	☐	☐
From the **mandatory hunter training course** bythe NYS Dept. of Environmental Conservation	☐	☐
From **optional hunting courses** bythe NYS Dept. of Environmental Conservation	☐	☐
From **online** resources or courses, **other than**a NYS Dept. of Environmental Conservation course	☐	☐
From an **in-person** course or workshop, **other than**a NYS Dept. of Environmental Conservation course	☐	☐
From local hunting clubs or groups	☐	☐
Other	☐	☐

38b Other - please describe:

____

38c Please specify online resources/courses and in-person courses used:

____

Page Break

39a Has anything prevented you from hunting or eating wild game as much as you wanted to?

- ◯ Yes
- ◯ No

39b **What prevented you from hunting (or eating wild game) as much as you wanted to?**

	Not a barrier	Minor barrier	Moderate barrier	Major barrier
Limited access to land and hunting opportunities	○	○	○	○
Time required to harvest and/or prepare wild game	○	○	○	○
Lack information about where to hunt	○	○	○	○
Lack skills required to hunt wild game	○	○	○	○
Lack skills required to process and prepare wild game meat	○	○	○	○
Lack people to hunt with and learn from	○	○	○	○
Concerns about impacting the environment	○	○	○	○
Concerns about safety of eating wild game	○	○	○	○
Cost of hunting license	○	○	○	○
Cost of hunting wild game (equipment, travel, etc.)	○	○	○	○
Don't like the act of killing an animal	○	○	○	○
Other	○	○	○	○

39c Other - please describe:

____

End of Block: Hunting and Wild Game

Start of Block: Fishing

Fishing_text You indicated that your household will fish or has fished this year. In this section we will ask about your fishing and angling practices.

Page Break

40 Which of the following best describes your fishing practices this year compared to last year?
I fished:

- ○ for the **first time** this year
- ○ **much more** this year
- ○ **a little more** this year
- ○ **the same** as last year
- ○ **a little less** this year
- ○ **much less** this year

Page Break

41a What types of fishing have you participated in? Please check all that apply.

	In 2019	Since the COVID-19 outbreak
Coldwater fishes (salmon, trout)	☐	☐
Warmwater fishes (bass, catfish, perch, sunfish, walleye, etc.)	☐	☐
Other	☐	☐

41b Other - please describe:

____

Page Break

42 **In the month you fished most**, about how many times did you go fishing?

- ◯ In 2019 (put # of times fished in the month you fished the most) ________________

- ◯ Since the COVID-19 outbreak(put # of times fished in the month you fished the most) ________________

43 How do you think COVID-19 will change your consumption of wild fish you caught compared to last year?
I will or have consumed wild fish I caught:

- ◯ for the **first time** this year
- ◯ **much more** this year
- ◯ **a little more** this year
- ◯ **the same**
- ◯ **a little less** this year
- ◯ **much less** this year

Page Break

44a How did/will you access the water for fishing? Check all that apply. **I access water from...**

	In 2019	Since the COVID-19 outbreak
Public boat ramps	☐	☐
Public water access by land	☐	☐
Public stream access	☐	☐
Land or water access I own	☐	☐
Privately owned land or access point I use for free	☐	☐
Privately owned land or access point I pay to use	☐	☐
A paid club membership	☐	☐
Other	☐	☐

44b Other - please describe:

____

Page Break

45a How did you get skills and knowledge for fishing? Check all that apply.

	In 2019	Since the COVID-19 outbreak
I already had skills and/or knowledge	☐	☐
From family/friends	☐	☐
From a course on fishing bythe NYS Dept. of Environmental Conservation	☐	☐
From online resources or courses, **other than** a NYS Dept. of Environmental Conservation course	☐	☐
From an in-person course or workshop, **other than** a NYS Dept. of Environmental Conservation course	☐	☐
From local fishing clubs or groups	☐	☐
Other	☐	☐

45b Other - please describe:

____

45c Please specify online resources/courses and in-person courses used:

____

Page Break

46a Has anything prevented you from fishing or angling as much as you wanted to?

- ◯ Yes
- ◯ No

46b **What has prevented you from fishing (or eating fish you catch) as much as you wanted to?**

	Not a barrier	Minor barrier	Moderate barrier	Major barrier
Limited access to water and fishing opportunities	○	○	○	○
Time required to catch and/or prepare fish	○	○	○	○
Lack information about where to catch or obtain fish	○	○	○	○
Lack skills required to catch fish	○	○	○	○
Lack skills required to process and prepare fish	○	○	○	○
Lack people to fish with and learn from	○	○	○	○
Concerns about impacting the environment	○	○	○	○
Concerns about safety of eating fish	○	○	○	○
Cost of fishing license	○	○	○	○
Cost of catching fish (equipment, travel, etc.)	○	○	○	○
Don't like the act of killing fish	○	○	○	○
Other	○	○	○	○

47c Other - please describe:

____

End of Block: Fishing

Start of Block: COVID-19 Impact on your Household

Impact_text In this section we will ask about the impact of COVID-19 on your job and food security.

Page Break

47 Have you or anyone in your household experienced a loss of income or job since the COVID-19 outbreak?

Check all that apply.

	Happened at all since the COVID-19 outbreak	Still happening today
Yes, lost job	○	○
Yes, reduced hours or income at job	○	○
Yes, furloughed	○	○
No, have not had any loss of job or income	○	○

End of Block: COVID-19 Impact on your Household

Start of Block: Food Security

48 Which of these statements best describes the food eaten in your household **in the last 12 months**:

○ Enough of the kinds of food I/we want to eat

○ Enough, but not always the kinds of food I/we want

○ Sometimes not enough to eat

○ Often not enough to eat

○ Don't know

○ Prefer not to answer

FS_text These next questions are about the food eaten in your household **in the last 30 days**, and whether you were able to afford the food you need.

You are going to read several statements that people have made about their food situation. For these statements, please indicate whether the statement was often true, sometimes true, or never true for your household in the last 30 days.

Page Break

49 "The food that my household bought just didn't last, and I/we didn't have the money to get more." Was that often, sometimes, or never true for your household **in the last 30 days**?

- ◯ Often true
- ◯ Sometimes true
- ◯ Never true
- ◯ Don't know

Page Break

50 "I/we couldn't afford to eat balanced meals." Was that often, sometimes, or never true for your household **in the last 30 days**?

- Often true
- Sometimes true
- Never true
- Don't know

Page Break

51 **In the last 30 days** did you or other adults in your household ever cut the size of your meals or skip meals because there wasn't enough money for food?

- ◯ Yes, almost every day
- ◯ Yes, some days but not every day
- ◯ Yes, only 1 or 2 days
- ◯ No
- ◯ Don't Know

Page Break

52 **In the last 30 days**, did you ever eat less than you felt you should because there wasn't enough money for food?

- ◯ Yes
- ◯ No
- ◯ Don't know

Page Break

53 **In the last 30 days**, were you very hungry but didn't eat because there wasn't enough money for food?

- Yes
- No
- Don't know

Page Break

FS_text **Now think about this same time last year**, the food eaten in your household, and whether you were able to afford the food you need.
For each statement, please indicate whether the statement was often true, sometimes true, or never true for you or your household at this time last year.

Page Break

54 "The food that my household bought just didn't last, and (I/we) didn't have the money to get more."

Was that often, sometimes, or never true for (you/your household) **at this time last year**?

- ◯ Often true
- ◯ Sometimes true
- ◯ Never true
- ◯ Don't know

Page Break

55 "(I/we) couldn't afford to eat balanced meals."

Was that often, sometimes, or never true for (you/your household) **at this time last year**?

- Often true
- Sometimes true
- Never true
- Don't know

Page Break

56 At this time last year, did you or other adults in your household ever cut the size of your meals or skip meals because there wasn't enough money for food?

- Yes, almost every day
- Yes, some days but not every day
- Yes, only 1 or 2 days
- No
- Don't Know

Page Break

57 **At this time last year**, did you ever eat less than you felt you should because there wasn't enough money for food?

- ◯ Yes
- ◯ No
- ◯ Don't know

Page Break

58 **At this time last year**, were you ever very hungry but didn't eat because there wasn't enough money for food?

- ◯ Yes
- ◯ No
- ◯ Don't know

End of Block: Food Security

Start of Block: Dietary Diversity/ Food Consumption

Diet_text In this section we will ask about what you have eaten recently.

Page Break

Diet_text **Think about what you usually ate in the past 3 months.**
Please think about all the fruits and vegetables, poultry, eggs, meat, and fish that you ate in the past 3 months. Include those that were:
1. Raw and cooked2. Eaten as snacks and at meals3. Eaten at home and away from home (restaurants, friends, take-out), and4. Eaten alone and mixed with other foods
Report how many times per month, week, or day you ate each food.
Choose the best answer for each question. Mark only one response for each question.

Page Break

59 Over the past 3 months, how many times have you eaten **fruits and vegetables from the following sources**?

	Never	1-3 times per month	1-2 times per week	3-4 times per week	5-6 times per week	1+ times per day
**Fruits and vegetables** from my garden	○	○	○	○	○	○
**Fruits and vegetables** grown by friends, family, and neighbors	○	○	○	○	○	○
**Fruits and vegetables** purchased from a farm or farmers' market (including CSAs)	○	○	○	○	○	○
**Fruits and vegetables** purchased from a store	○	○	○	○	○	○

Page Break

60 Over the past 3 months, how many times have you eaten **eggs from the following sources?**

	Never	1-3 times per month	1-2 times per week	3-4 times per week	5-6 times per week	1+ times per day
**Eggs** from poultry that I raised	o	o	o	o	o	o
**Eggs** from poultry raised by friends, family, and neighbors	o	o	o	o	o	o
**Eggs** purchased from a farm or farmers' market	o	o	o	o	o	o
**Eggs** purchased in a store	o	o	o	o	o	o

Page Break

61 Over the past 3 months, how many times have you eaten **poultry from the following sources?**

	Never	1-3 times per month	1-2 times per week	3-4 times per week	5-6 times per week	1+ times per day
**Poultry** that I raised	○	○	○	○	○	○
**Poultry** raised by friends, family, and neighbors	○	○	○	○	○	○
**Poultry** purchased from a farm or farmers' market	○	○	○	○	○	○
**Poultry** purchased in a store	○	○	○	○	○	○

Page Break

62 Over the past 3 months, how many times have you eaten **foraged foods from the following sources?** Note: **Foraging** means searching for and harvesting wild produce, including berries or other fruits, greens or other vegetables, roots, mushrooms, and medicinal plants.

	Never	1-3 times per month	1-2 times per week	3-4 times per week	5-6 times per week	1+ times per day
**Foods foraged** by me	○	○	○	○	○	○
**Foods foraged** by friends, family, or neighbors	○	○	○	○	○	○
**Foraged foods** purchased from a farm or farmers' market	○	○	○	○	○	○

Page Break

63 Over the past 3 months, how many times have you eaten **fish or seafood from the following sources?**

	Never	1-3 times per month	1-2 times per week	3-4 times per week	5-6 times per week	1+ times per day
**Fish or seafood** that I caught	○	○	○	○	○	○
**Fish or seafood** caught by friends, family, or neighbors	○	○	○	○	○	○
**Fish and seafood** that was purchased	○	○	○	○	○	○

Page Break

64 Over the past 3 months, how many times have you eaten **wild game from the following sources?**

	Never	1-3 times per month	1-2 times per week	3-4 times per week	5-6 times per week	1+ times per day
**Wild game** that I harvested	○	○	○	○	○	○
**Wild game** harvested by friends, family, or neighbors	○	○	○	○	○	○
**Wild game** from a food pantry or food bank	○	○	○	○	○	○

Page Break

65 **Compared to this time last year,** how often have you been eating the following items in the past 3 months?
Please compare the months of July - September between last year (2019) and this year (2020).

	Much more	A little more	About the same	A little less	Much less
Fruits and vegetables	○	○	○	○	○
Eggs	○	○	○	○	○
Poultry (like chicken, turkey, etc.)	○	○	○	○	○
Foraged foods	○	○	○	○	○
Purchased fish or seafood	○	○	○	○	○
Locally harvested wild fish	○	○	○	○	○
Hunted wild game	○	○	○	○	○
Processed and red meats	○	○	○	○	○

End of Block: Dietary Diversity/ Food Consumption

Start of Block: Background Demographics

Demo_text In this final section we will ask about who the people in your household are.

Page Break

66 How many people in the following age groups currently live in your household (including family and non-family members)?

Adults over 65	▼ 0 ... 7+
Adults 18-65	▼ 0 ... 7+
Children 5-17	▼ 0 ... 7+
Children under 5	▼ 0 ... 7+

Page Break

67 What is the highest level of formal education that you have completed?

- ◯ Some high school (no diploma)
- ◯ High school graduate (including GED)
- ◯ Some college (no degree)
- ◯ Associates degree/technical school/apprenticeship
- ◯ Bachelor’s degree
- ◯ Postgraduate (like Master’s, PhD) / professional degree (like JD)

Page Break

68 Which of the following best describes your household income range in 2019 before taxes?

- < $15,000
- $15,000 to $24,999
- $25,000 to $49,999
- $50,000 to $74,999
- $75,000 to $149,999
- $150,000 or more

Page Break

69 Which of the following best describes your gender identity?

- ◯ Male
- ◯ Female
- ◯ Prefer to self-describe: ____
- ◯ Prefer not to answer

Page Break

70 With which racial and ethnic groups do you identify? Check all that apply:

- ☐ American Indian or Alaska Native
- ☐ Asian or Asian American
- ☐ Black or African American
- ☐ Hispanic, Latinx, or Spanish origin
- ☐ Middle Eastern or North African
- ☐ Native Hawaiian or Pacific Islander
- ☐ White
- ☐ Not listed here or prefer to self-describe: ____________________
- ☐ Prefer not to answer

Page Break

71 Are you of Hispanic, Latino, or Spanish origin?

- ◯ No, not of Hispanic, Latino, or Spanish origin
- ◯ Yes, Mexican, Mexican American, Chicano
- ◯ Yes, Puerto Rican
- ◯ Yes, Cuban
- ◯ Yes, another Hispanic, Latino, or Spanish origin: ____

Page Break

72 Which of the following political affiliations do you most identify with?

- ◯ Republican
- ◯ Democrat
- ◯ Independent
- ◯ No affiliation
- ◯ Other

End of Block: Background Demographics

Start of Block: Wrap Up

73 Thank you for completing the survey!
If you would like, please share any other comments or experiences harvesting or accessing food, whether related or unrelated to COVID-19:

____

____

____

____

____

74a May we contact you to learn more about the topics in this survey?

- ◯ Yes
- ◯ No

74b Thank you!

We look forward to talking with you. So we can reach you, please provide your email address and/or phone number here:

- ○ Email address ____

- ○ Phone number ____

Page Break

75a Would you like to enter the raffle for survey completion? Your contact information will NOT be associated with your survey answers.

- ○ Yes
- ○ No

End of Block: Wrap Up
