# Supplementary material for: Wild and backyard food use during COVID-19 in upstate New York, United States
Source: Front Nutr. 2023 Sep 5;10:1222610. doi: 10.3389/fnut.2023.1222610 (PMC10507697; doi:10.3389/fnut.2023.1222610)
Supplement: Supplementary file 2 [file Data_Sheet_2.docx]

Supplemental Material

Table 1: Selected demographics percentages compared to 2015 census of sampled counties

|  | Census % | Sample % |
| --- | --- | --- |
| **Race** | | |
| White | 88.4 | 91.4 |
| Black | 4.3 | 1.2 |
| Native American | 0.3 | 1.6 |
| Asian | 1.7 | 1.4 |
| Hispanic or Latino | 3.2 | 2.6 |
| Multiracial | 4.1 | NA |
| **Gender** | | |
| Male | 48.1 | 31.6 |
| Female | 48.0 | 63.6 |
| Other* | 4.0 | 4.9 |

*categories for race, age, and gender are as written in the 2015 USA Census

Table 2: Associations between pandemic-related challenges and increased wild and backyard food consumption.

|  | **Food Secure (%)** | **Food Insecure (%)** | **p** | **No Food Assistance (%)** | **Any Food Assistance (%)** | **p** | **No Income Loss (%)** | **Income loss (%)** | **p** |
| --- | --- | --- | --- | --- | --- | --- | --- | --- | --- |
| Fruit & Vegetables | 32.4 | 35.8 | 0.71 | 30.0 | 41.7 | 0.80 | 31.1 | 36.8 | 0.31 |
| Eggs | 2.0 | 5.7 | 1.0 | 1.8 | 4.2 | 1.0 | 1.9 | 3.5 | 0.12 |
| Poultry | 3.3 | 0 | 0.21 | 1.8 | 6.7 | 1.0 | 2.8 | 3.5 | 0.60 |
| Foraged Foods | 10.1 | 15.1 | 0.67 | 8.5 | 17.5 | 0.12 | 10.2 | 11.8 | 0.15 |
| Fish or Seafood | 8.8 | 9.4 | 0.39 | 7.5 | 13.3 | 0.43 | 8.3 | 10.4 | 1.0 |
| Wild Game | 9.0 | 15.1 | 0.57 | 8.0 | 15.0 | 0.49 | 9.4 | 10.4 | 0.41 |
